# Mass cytometry analysis reveals altered immune profiles in patients with coronary artery disease

**DOI:** 10.1002/cti2.1462

**Published:** 2023-11-02

**Authors:** Katharine A Kott, Adam S Chan, Stephen T Vernon, Thomas Hansen, Taiyun Kim, Macha de Dreu, Bavani Gunasegaran, Andrew J Murphy, Ellis Patrick, Peter J Psaltis, Stuart M Grieve, Jean Y Yang, Barbara Fazekas de St Groth, Helen M McGuire, Gemma A Figtree

**Affiliations:** ^1^ Cardiothoracic and Vascular Health Kolling Institute of Medical Research Sydney NSW Australia; ^2^ Department of Cardiology, Royal North Shore Hospital Northern Sydney Local Health District Sydney NSW Australia; ^3^ Northern Clinical School, Faculty of Medicine and Health University of Sydney Sydney NSW Australia; ^4^ School of Mathematics and Statistics University of Sydney Sydney NSW Australia; ^5^ Charles Perkins Centre University of Sydney Sydney NSW Australia; ^6^ School of Medical Sciences, Faculty of Medicine and Health University of Sydney Sydney NSW Australia; ^7^ Baker Heart and Diabetes Institute Melbourne VIC Australia; ^8^ Monash Cardiovascular Research Centre Clayton VIC Australia; ^9^ Department of Radiology Royal Prince Alfred Hospital Sydney NSW Australia; ^10^ Imaging and Phenotyping Laboratory, Charles Perkins Centre, Faculty of Medicine and Health University of Sydney Sydney NSW Australia; ^11^ Ramaciotti Facility for Human Systems Biology University of Sydney Sydney NSW Australia

**Keywords:** atherosclerosis, immune signature, inflammation, mass cytometry, T regulatory cells

## Abstract

**Objective:**

The importance of inflammation in atherosclerosis is well accepted, but the role of the adaptive immune system is not yet fully understood. To further explore this, we assessed the circulating immune cell profile of patients with coronary artery disease (CAD) to identify discriminatory features by mass cytometry.

**Methods:**

Mass cytometry was performed on patient samples from the BioHEART‐CT study, gated to detect 82 distinct cell subsets. CT coronary angiograms were analysed to categorise patients as having CAD (CAD^+^) or having normal coronary arteries (CAD^−^).

**Results:**

The discovery cohort included 117 patients (mean age 61 ± 12 years, 49% female); 79 patients (68%) were CAD^+^. Mass cytometry identified changes in 15 T‐cell subsets, with higher numbers of proliferating, highly differentiated and cytotoxic cells and decreases in naïve T cells. Five T‐regulatory subsets were related to an age and gender‐independent increase in the odds of CAD incidence when expressing CCR2 (OR 1.12), CCR4 (OR 1.08), CD38 and CD45RO (OR 1.13), HLA‐DR (OR 1.06) and Ki67 (OR 1.22). Markers of proliferation and differentiation were also increased within B cells, while plasmacytoid dendritic cells were decreased. This combination of changes was assessed using SVM models in discovery and validation cohorts (area under the curve = 0.74 for both), confirming the robust nature of the immune signature detected.

**Conclusion:**

We identified differences within immune subpopulations of CAD^+^ patients which are indicative of a systemic immune response to coronary atherosclerosis. This immune signature needs further study *via* incorporation into risk scoring tools for the precision diagnosis of CAD.

## Graphical abstract

Atherosclerosis, arguably the largest cause of human morbidity and mortality, is progressively being accepted as a chronic inflammatory disease. Here, we used mass cytometry to perform an assessment of the differences in peripheral immune cell populations in patients with CT coronary angiogram‐defined coronary artery disease (CAD), and identified 18 immune subsets that were related to the disease phenotype. CAD patients had lower numbers of naïve CD4 and CD8 T cells, higher proliferation of T regulatory cells and B cells, higher numbers of differentiated conventional and regulatory CD4 T cells and increased terminally differentiated CD4 and CD8 T cells with the cytotoxic KLRG1^+^CD27^‐^ phenotype, which we demonstrate improves the predictive ability of atherosclerotic risk models.

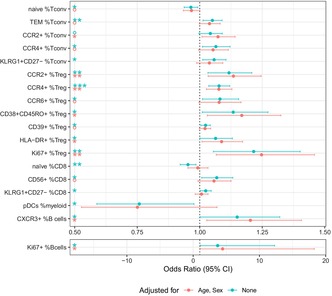

## Introduction

Coronary artery disease (CAD) remains a major cause of global disease burden^1^ despite widespread identification and treatment of the standard modifiable risk factors (SMuRFs). Recent studies have shown an increasing proportion of CAD patients presenting with acute coronary syndrome who do not have any of these risk factors.^2^, ^3^, ^4^ While efforts to mitigate known causes of disease should continue to be optimised, it is likely that important disease mechanisms and treatment targets remain to be discovered, potentially conferring broad benefits to all patients with CAD.

The clinical importance of the links between atherosclerosis and inflammation were initially confirmed by the Canakinumab Anti‐inflammatory Thrombosis Outcome Study (CANTOS) trial,^5^ which showed a reduction in recurrent cardiovascular events after treatment with a monoclonal antibody targeting interleukin‐1β. While the application of such a broad anti‐inflammatory therapy resulted in an increase in fatal infection that limits clinical utility, promising results have also been reported from studies researching anti‐inflammatory therapy with colchicine in those with chronic coronary disease^6^ and following myocardial infarction.^7^ This marks the beginning of a new line of potential therapies for CAD. Increasing our knowledge of the involvement of the immune system in atherosclerosis will improve our ability to identify precision drug targets in the future.

Cardioimmunology focuses on the dynamic relationship between the heart and the multitude of immune cells involved in cardiac homeostasis and disease.^8^ In recent decades, research in this area has expanded greatly and we have recently reviewed the findings from modern single‐cell studies relating immune cell types to atherosclerosis.^9^ At time of writing, the major single‐cell study assessing atherosclerosis in humans is by Fernandez *et al*.,^10^ who in 2019 characterised the immune cells present in excised carotid atheroma and compared this with the circulating immune profile using a combination of single‐cell techniques. As would be expected in a comparison of tissue *vs* circulating T cells, this study reported that T cells located in plaque were more activated, differentiated and showed more exhaustion markers when compared to their circulating counterparts. Other studies have shown that circulating peripheral monocyte, NK and T‐cell subsets are altesred in CAD in humans,^11^, ^12^, ^13^, ^14^, ^15^, ^16^, ^17^ though these have largely been flow cytometry‐based analyses using only a limited number of parameters.

In cardiology, unlike oncology, it is not routine to perform biopsies to provide insight into the specific nature of the diseased tissue present in a given individual. To further the progress of precision medicine strategies in CAD, we need to develop diagnostic techniques that utilise a combination of advanced imaging techniques and analysis of non‐invasive biological samples to further our understanding of the disease state and the host response to that disease. To inform this future work, we analysed peripheral mononuclear blood cells (PBMCs) from patients with computed tomography coronary angiography (CTCA) quantified coronary artery disease. We employed the emerging technology of mass cytometry which allows for metal‐conjugated antibody‐based tagging of intracellular and surface proteins using spectrophotometric discrimination, resulting in the ability to simultaneously evaluate a much higher number of cell subtypes than is possible in flow cytometry. Mass cytometric analysis of PBMCs has been used previously to identify immune cell profiles associated with specific disease phenotypes,^18^, ^19^, ^20^ and we hypothesised that circulating immune cells would also reflect the immune processes responsible for atherosclerosis at the level of the vessel wall. To assess this, we performed mass cytometry analysis with a broad panel of markers to determine the differences present in those with and without CAD.

## Results

The clinical characteristics of the discovery cohort are presented in Table 1. Of the 117 patients, 79 (68%) had CAD. On average, the CAD^+^ patients were older (64 ± 10 years *vs* 53 ± 13 years, *P* < 0.001), more likely to be male (59% *vs* 34%, *P* = 0.010) and to have hypertension (51% *vs* 26%, *P* = 0.013) and hyperlipidaemia (60% *vs* 40%, *P* = 0.042). The CAD^+^ patients were also more likely to be receiving statin therapy (39% *vs* 18%, *P* = 0.024), and ACE inhibitor or angiotensin receptor blocker therapy (42% *vs* 21%, *P* = 0.028). There were fewer ‘SMuRFless’ patients who had none of the traditional modifiable risk factors in the CAD^+^ group (14% *vs* 32%, *P* = 0.024). There were no significant differences between the groups in terms of BMI, diabetes, smoking (current or significant history), family history, use of antiplatelet agents or use of beta‐blockers. A Gensini score of zero defined the CAD^−^ group, and the median Gensini score in the CAD^+^ group was 7.5, consistent with early atherosclerosis.

**Table 1:** Discovery cohort characteristics

Demographics, risk factors and medications	All *n* = 117	CAD^−^ *n* = 38	CAD^+^ *n* = 79	*P*
Sex, male – *n* (%)	57 (49%)	13 (34%)	47 (59%)	0.010
Age, years – mean (SD)	61 (12)	53 (13)	64 (10)	< 0.001
BMI, kg/m^2^ – mean (SD)	27.1 (4.8)	27.0 (5.3)	27.1 (4.7)	0.909
Hypertension – *n* (%)	50 (43%)	10 (26%)	40 (51%)	0.013
Diabetes – *n* (%)	8 (7%)	2 (5%)	6 (8%)	0.640
Hyperlipidaemia – *n* (%)	62 (53%)	15 (40%)	47 (60%)	0.042
Current smoking – *n* (%)	13 (11.1%)	5 (13%)	8 (10%)	0.625
Significant smoking history (> 10 pack years) – *n* (%)	23 (20%)	5 (13%)	18 (23%)	0.220
Patients with no major risk factors (SMuRFless) – *n* (%)	23 (20%)	12 (32%)	11 (14%)	0.024
Significant family history of ischaemic heart disease – *n* (%)	33 (28%)	11 (29%)	22 (28%)	0.902
Antiplatelet – *n* (%)	23 (20%)	8 (21%)	15 (19%)	0.792
Statin – *n* (%)	38 (33%)	7 (18%)	31 (39%)	0.024
Beta‐blocker – *n* (%)	19 (16%)	5 (13%)	14 (18%)	0.531
ACE inhibitor/Angiotensin Receptor Blocker – *n* (%)	41 (35%)	8 (21%)	33 (42%)	0.028
Gensini score – median (IQR)	‐	‐	7.5 (13.5)	‐

ACE, angiotensin converting enzyme; BMI, body mass index; CAD, coronary artery disease; IQR, interquartile range; SD, standard deviation; SMuRFs, standard modifiable risk factors.

*P*‐values were calculated using the Wilcoxon rank sum test for continuous variables; Fisher's exact test for categorical variables with expected cell counts < 5; and Pearson's Chi‐squared test for categorical variables with expected cell count ≥ 5.

### Approach to analysis of mass cytometry data

To comprehensively address whether differences in circulating immune subsets align with cardiovascular disease, we analysed peripheral blood samples using the Helios mass cytometry platform. A 41‐parameter antibody panel allowed us to employ an analysis strategy of hierarchical sequential gating, capturing the complex but well‐described immunobiological relationships defined by differential marker expression. For all major immune populations (i.e. T conventional and T regulatory CD4^+^ T cells, CD8^+^ T cells and B cells), we analysed the expression of every marker and then discarded non‐informative analyses. The net result was identification of 82 distinct immune cell subsets within 11 major immune populations, outlined in Supplementary figures 1–3. This analysis approach has the advantage of seamless future translation into a clinical setting in which clearly defined immune subpopulations can be identified using conventional techniques. In contrast, semi‐automatic or automatic analysis may identify populations that do not readily map to conventionally defined immune cell subtypes. Importantly, we confirmed that our traditional gating strategy captured all 11 major immune populations by applying an unsupervised dimensionality reduction approach, UMAP visualisation (Supplementary figure 4a, b). An exploration of the residual cells not included in the 11 major populations confirmed that the majority were cell doublets that had been excluded by our hierarchical sequential gating (Supplementary figure 4c).

### Association of major cell subsets with age, sex or CAD status

**Figure 1:**
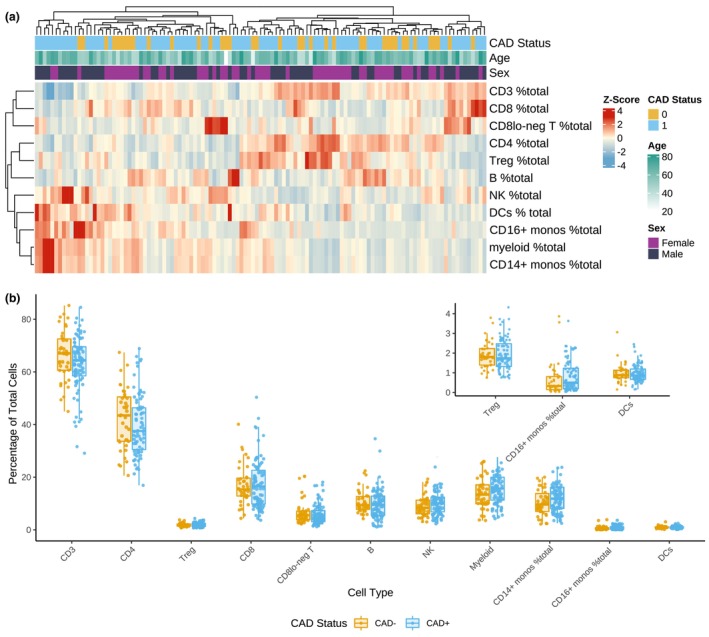
**(a)** Heat map of the cell proportions in patients clustered *via* hierarchical clustering with Euclidean distance and complete linkage; *n* = 177. **(b)** Boxplots illustrating the distribution of the percentage of total live cells (*y*‐axis) for major immune cell subtypes (*x*‐axis) between non‐diseased (CAD^−^) and diseased (CAD^+^) individuals; *n* = 177.

Unsupervised hierarchical clustering analysis of the distribution of principal blood cell types, gated as per Supplementary figures 1–3 and expressed as a proportion of total live cells, indicated that there were no major effects identified for age, sex or CAD status, as presented *via* a heat map in Figure 1a. Comparisons of the proportions of major cell types within PBMCs between the CAD^−^ and CAD^+^ groups are shown in Figure 1b. Logistic regression analysis identified no significant differences in the numbers of any major cellular subtype when expressed as a percentage of total live cells.

### Association of conventional CD4 T‐cell subpopulations with CAD

**Figure 2:**
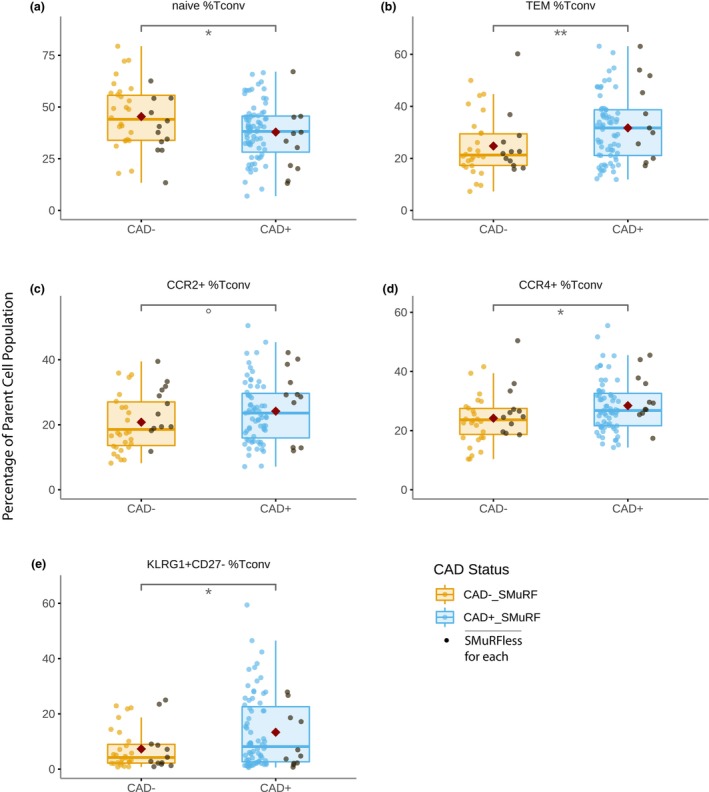
Boxplots showing the proportions of **(a)** naive, **(b)** TEM, **(c)** CCR2^+^, **(d)** CCR4^+^ and **(e)** KLRG1^+^CD27^-^ conventional CD^4^ T cells (Tconv) comparing non‐diseased (CAD^−^) and diseased (CAD^+^) individuals, with a red diamond indicating the group mean. Patients who had no standard risk factors (SMuRFless) shown in black. *P*‐values were calculated as significance of coefficient of each population in logistic regression models; *n* = 177. °*P* < 0.10 *P < 0.05, **P < 0.01.

To determine whether CAD was associated with specific cellular subsets within the major cell types, we performed analysis using the gating strategy outlined in Supplementary figures 1–3, (with example plots demonstrating relatively ‘low’ and ‘high’ proportions, in panel b of each supplementary figure). The strategy made use of combinations of cellular markers to improve the precision of subset identification. For example, CD45RA, CD45RO, CCR7 and CD27 were all included in the gating tree to define T cells in the naïve, central memory (TCM), effector memory (TEM) and terminally differentiated effector memory (TEMRA) compartments. Patients with CAD had a significantly different distribution of cellular subpopulations within conventional (non‐regulatory) CD4 T cells (Tconv), with a decrease in naïve cells and a reciprocal increase in differentiated cells compared to patients with no CAD (Figure 2). These differences were seen in subjects irrespective of whether they had standard modifiable risk factors (SMuRFs) or not. CAD was associated with a significant decrease in the proportion of naïve Tconv cells [37.9% ± 14.0% *vs* 45.4% ± 15.4%, *P* = 0.013] and an increase in TEM [31.7% ± 12.9% *vs* 24.7% ± 11.6%, *P* = 0.008]. The proportion of CD4 Tconv cells expressing the chemokine receptor 4 (CCR4) was also increased [28.4% ± 8.7% *vs* 24.2% ± 8.6%, *P* = 0.018]. Initial analysis indicated an increase in the heterogenous population of CD4 Tconv expressing killer cell lectin‐like receptor G1 (KLRG1). Further analysis indicated that this increase was restricted to the highly differentiated subset of KLRG1‐expressing cells that no longer expressed CD27 [13.4% ± 13.0% *vs* 7.3% ± 7.4%, *P* = 0.013]. The difference in the proportion of Tconv expressing chemokine receptor 2 (CCR2) did not reach statistical significance at the 0.05 level [24.2% ± 9.5% *vs* 20.8% ± 8.7%, *P* = 0.069] without adjustment for age and sex (see Logistic Regression Models below).

### Association of T regulatory cell subpopulations with CAD

**Figure 3:**
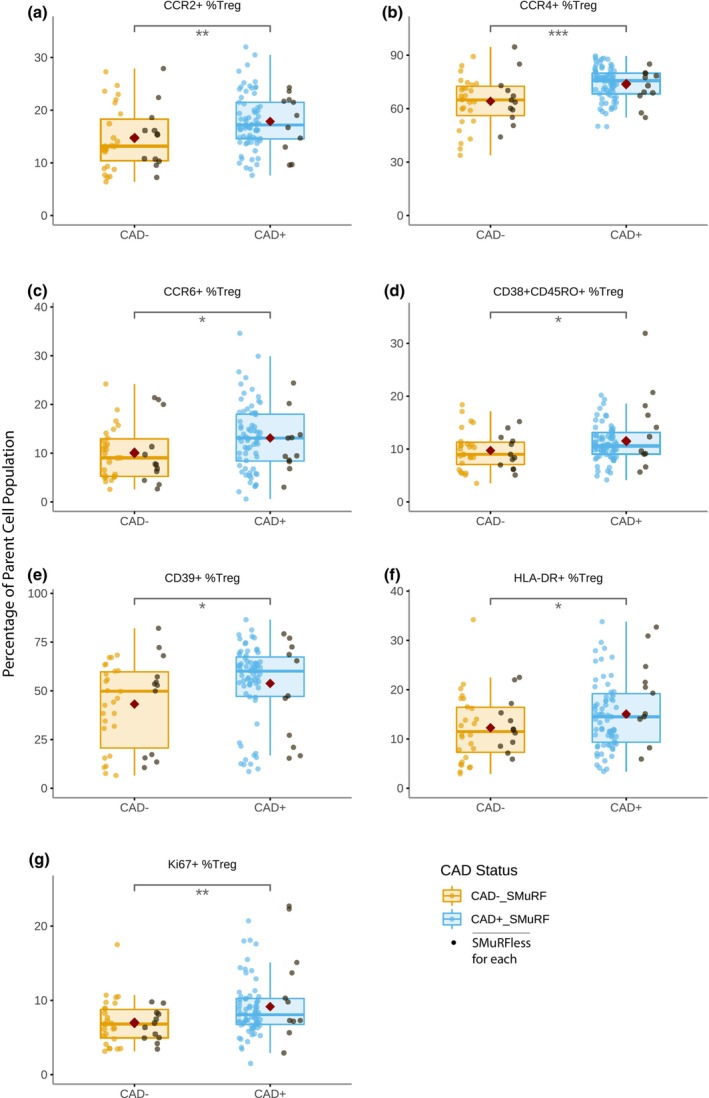
Boxplots showing the proportions of **(a)** CCR2^+^, **(b)** CCR4^+^, **(c)** CCR6^+^, **(d)** CD38^+^CD45RO^+^, **(e)** CD39^+^, **(f)** HLA‐DR^+^, and **(g)** Ki67^+^ T regulatory cells (Treg) comparing non‐diseased (CAD^−^) and diseased (CAD^+^) individuals, with a red diamond indicating the group mean. Patients who had no standard risk factors (SMuRFless) shown in black. *P*‐values were calculated as significance of coefficient of each population in logistic regression models; *n* = 177. *P < 0.05, **P < 0.01, ***P < 0.001.

We next examined specific subpopulations of T regulatory cells (Tregs) associated with CAD (Figure 3). The CAD group had a significantly higher proportion of Tregs expressing the chemokine receptors CCR2 [17.9% ± 5.3% *vs* 14.7% ± 5.9%, *P* = 0.006], CCR4 [73.7% ± 9.2% *vs* 64.1% ± 14.1%, *P* < 0.001] and CCR6 [13.1% ± 6.8% *vs* 10.1% ± 5.7%, *P* = 0.022]. Cells co‐expressing CD38 and CD45RO [11.5% ± 4.3% *vs* 9.7% ± 3.6%, *P* = 0.033], CD39 [53.8% ± 20.5% *vs* 43.2% ± 21.6%, *P* = 0.014], HLA‐DR [15.1% ± 7.1% *vs* 12.3% ± 6.7%, *P* = 0.049] and Ki67 [9.2% ± 4.2% *vs* 7.0% ± 2.9%, *P* = 0.007] were also significantly increased in the CAD^+^ group.

Once again, the differences between the CAD^−^ and CAD^+^ groups were similar in subjects with no SMuRFs. We performed additional analysis of the Treg subpopulations to determine whether the CAD differences observed were being driven by significant differences in use of ACE inhibitors/angiotensin receptor blockers (ARBs) or statins (Table 1). Neither treatment with ACE inhibitors/ARBs nor statins had a major impact on the proportion of Treg subpopulations of interest (Supplementary figure 5).

Given the striking CAD associations across Treg subsets as identified by single marker expression, we explored marker co‐expression within these Treg subsets by looking at the proportion of cells allocated to more than one subset. Supplementary figure 1c shows two distinct patterns of co‐expression: association of CD39^+^ and CCR4^+^, both indicative of heightened Treg suppressive function,^21^, ^22^ and association of Ki67^+^ and HLA‐DR^+^, a phenotype attributable to recent activation of cells.^23^ None of the other Treg markers associated with CAD were found to be significantly co‐expressed.

### Association of CD8 T‐cell subpopulations with CAD

**Figure 4:**
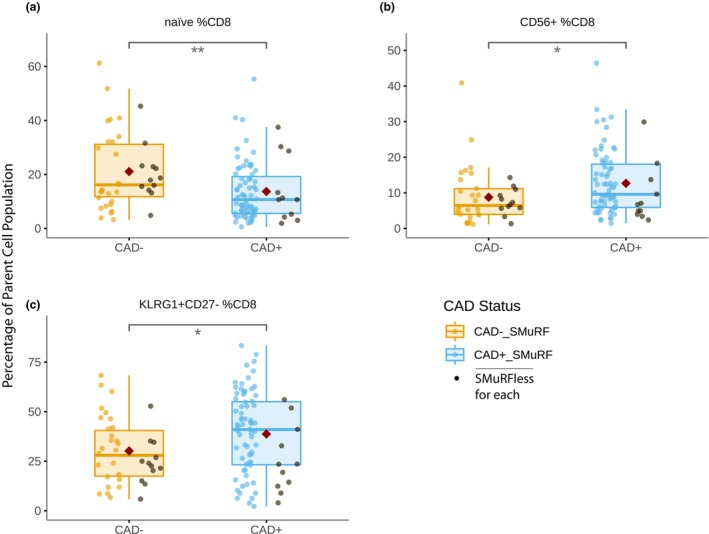
Boxplots showing the proportions of **(a)** naive, **(b)** CD56^+^ and **(c)** KLRG1^+^CD27^-^ CD8 T cells comparing non‐diseased (CAD^−^) and diseased (CAD^+^) individuals, with a red diamond indicating the group mean. Patients who had no standard risk factors (SMuRFless) shown in black. *P*‐values were calculated as significance of coefficient of each population in logistic regression models; *n* = 177. *P < 0.05, **P < 0.01.

Within CD8 T cells (Figure 4), CAD was associated with a significant decrease in the proportion of naïve cells [13.7% ± 10.7% *vs* 21.1% ± 14.2%, *P* = 0.004] and an increase in cells expressing CD56 [12.7% ± 8.9% *vs* 8.8% ± 7.6%, *P* = 0.026]. Similar to CD4 Tconv, the CAD‐associated increase in KLRG1 expression in CD8 T cells was limited to the cytotoxic KLRG1^+^CD27^−^ subset [38.7% ± 20.5% *vs* 30.2% ± 16.4%, *P* = 0.030], and again these changes were present in both the SMuRFless subjects and in those who had traditional CAD risk factors.

### Association of non‐T‐cell subpopulations with CAD

**Figure 5:**
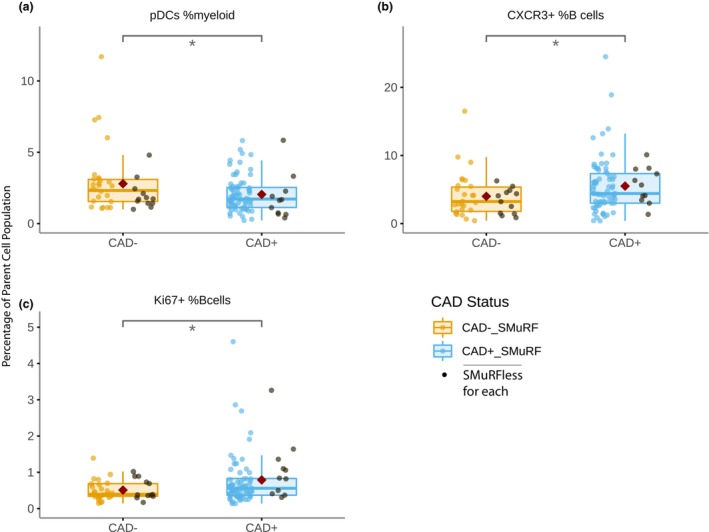
Boxplots showing the proportions of **(a)** proliferating CD16^+^CD56^int^ NK cells and **(b, c)** proliferating B cells, comparing non‐diseased (CAD^−^) and diseased (CAD^+^) individuals, with patients who had no standard risk factors (SMuRFless) shown in black. *P*‐values were calculated as significance of coefficient of each population in logistic regression models; *n* = 177. *P < 0.05.

Subtypes within B cells, natural killer (NK) cells, monocytes and dendritic cells were also identified using the gating strategy outlined in Supplementary figure 3. Only three subpopulations of non‐T cells were significantly different in those with disease, irrespective of SMuRF status (Figure 5). Plasmacytoid dendritic cells (pDCs) were significantly reduced in the CAD^+^ group [2.0% ± 1.3% *vs* 2.8% ± 2.1%, *P* = 0.032] while B cells expressing CXCR3 [5.5% ± 4.0% *vs* 4.0% ± 3.1%, *P* = 0.048] and Ki67 [0.8% ± 0.7% *vs* 0.5% ± 0.3%, *P* = 0.033] were increased. While no specific monocyte differences were identified, the analysis revealed suboptimal CD16 marker detection resulting in a notably atypical population, so the results are unlikely to reliably indicate the diversity of the blood monocyte populations.

### Logistic regression models

**Figure 6:**
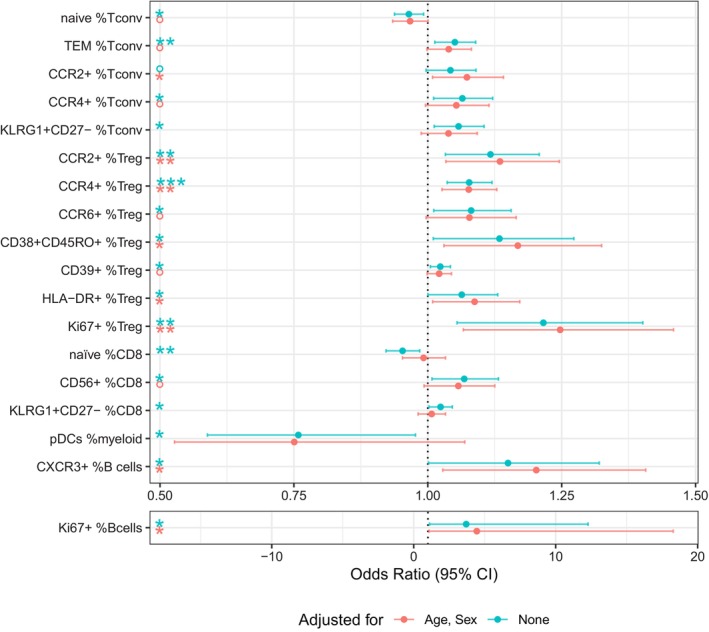
Forest plot demonstrating the odds of increase in coronary artery disease (CAD) incidence for every unit increase in the cell subset. *P*‐values were calculated as significance of coefficient of each population in logistic regression models; *n* = 177. °*P* < 0.1, *P < 0.05, **P < 0.01, ***P < 0.001.

To examine the effect size and the potential confounding impact of age and sex on the associations of CAD with specific cell subpopulations, we performed regression analyses on the 15 T‐cell subpopulations described in Figures 2, 3, 4 plus the 3 non‐T‐cell populations shown in Figure 5. Figure 6 shows unadjusted odds ratios contrasted with odds ratios adjusted for age and sex. The odds ratio indicates the increase in the odds of CAD incidence for every unit increase in the cell subset percentage.

Within the CD4 Tconv subsets, only the CCR2^+^ subgroup was significantly different in the CAD^+^ group after adjustment, with an odds ratio of 1.07 (95% CI: 1.01–1.14, *P* = 0.025). Five of the seven Treg subsets remained significant after adjustment, with odds ratios for CCR2^+^ of 1.13 (95% CI: 1.03–1.25, *P* = 0.008), CCR4^+^ of 1.08 (95% CI: 1.03–1.13, *P* = 0.002), CD38^+^CD45RO^+^ of 1.17 (95% CI: 1.03–1.32, *P* = 0.015), HLA‐DR^+^ of 1.09 (95% CI: 1.01–1.17, *P* = 0.028) and Ki67^+^ of 1.25 (95% CI: 1.07–1.46, *P* = 0.006). None of the CD8 subsets remained significant in the adjusted model nor did pDCs. Both B‐cell subsets remained significant after adjustment, with odds ratios for CXCR3^+^ of 1.20 (95% CI: 1.03–1.41, *P* = 0.021) and Ki67^+^ of 4.44 (95% CI: 1.09–18.28, *P* = 0.039). This analysis indicates that while age and sex adjustment had only a minor effect on the odds ratio for Tregs and B cells, the effects on the CD4 Tconv and CD8 T‐cell subsets were larger, suggesting that the differences in conventional T‐cell subsets between the CAD^+^ and CAD^−^ groups were likely because of the older age of the CAD^+^ group.

### Independent validation and modelling confirms robustness of results

**Figure 7:**
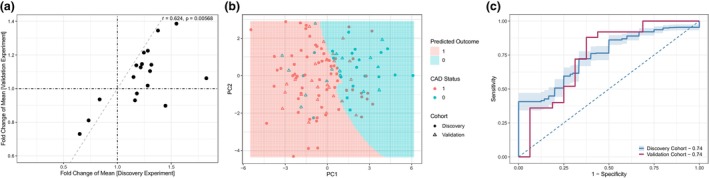
**(a)** Correlation of results between the discovery (*n* = 177) and validation (*n* = 58) experiments, shown as fold difference of means, *P*‐value calculated from test for correlation. **(b)** Decision boundary plot of the patients over age 55 (*n* = 84) using the first two principal components of the CyTOF data coloured by coronary artery disease (CAD) status, overlaid with the predicted CAD status from the model trained on the discovery cohort only. **(c)** A plot of the model performance from a repeated five‐fold cross‐validation with 20 repeats, showing the average receiver‐operator curve curve and AUC values for both discovery (*n* = 177) and validation (*n* = 58) experiments.

To confirm these results, a validation experiment of 58 additional patients was analysed by mass cytometry and gated for the same subpopulations. The clinical and demographic characteristics of this group are presented in Supplementary table 2. To account for batch effects that prevented direct comparisons between the experiments, we examined the fold change in results for CAD^+^ and CAD^−^ groups between the discovery cohort and the validation cohort (Figure 7a). In the validation experiment, the fold changes were generally lower compared to the discovery cohort, but the trend was retained for 12 of the 15 increased populations, and for all 3 of the decreased populations. Overall, there was a significant concordance between the two sets of results (*r* = 0.624, *P* = 0.006).

To identify a predictive CyTOF signature, we built a number of risk models. Initial lasso modelling of the whole discovery cohort produced an average AUC of 0.65 (Supplementary figure 6a). Adapting a multi‐step classification concept,^24^ we identified age as the modifying variable that was most associated with the classifiability of patients. This enabled us to identify a sub‐cohort of patients aged 55 or greater (Supplementary table 3 and Supplementary Figure 6b) where the prognostic ability of the CyTOF signature was strongest. We then applied a radial kernel SVM model to the 18 variables from the CyTOF analysis in the discovery cohort for subjects aged 55 and above. The decision boundary plot demonstrates that the majority of the points were correctly classified (Figure 7b). The model performance for the CyTOF signature alone in the discovery and validation cohorts is shown as a receiver‐operator curve displayed in Figure 7c, with the same average AUC for the discovery cohort (AUC = 0.74) and the validation cohort (AUC = 0.74). In comparison, models containing only age and sex alone had lower AUCs for both discovery (AUC = 0.73) and validation (AUC = 0.69) cohorts. Combining the CyTOF signature with age and sex generated the highest AUCs for both discovery (AUC = 0.76) and validation (AUC = 0.79) cohorts.

## Discussion

Here, we have systematically determined the proportions of 82 immune cell subsets within the PBMCs of 117 patients who have had CTCAs assessed for the presence or absence of CAD in the largest CyTOF‐based study of human coronary atherosclerosis to date. Our comprehensive hierarchical gating strategy (validated by unsupervised dimensionality reduction techniques) faithfully recapitulated immunobiological relationships, to demonstrate real differences in clearly defined cell subpopulations. Most notably, we have demonstrated that CAD is associated with higher proportions of T regulatory cells expressing CCR2, CCR4, CCR6, CD39, HLA‐DR, Ki67 and the combination of CD38 and CD45RO, and shown that this association remains significant after adjustment for age and sex. These findings were verified in an independent validation experiment, which demonstrated concordance across a second cohort of 58 patients.

The role of T cells in atherosclerosis has recently been reviewed in detail,^25^ but few studies have used high‐dimensional cytometry to assess changes in the circulating lymphocyte profile in association with CAD. Interestingly, our detailed analysis of multiple T‐cell and non‐T‐cell subsets showed that changes associated with coronary atherosclerosis were primarily identified within T‐cell subpopulations and were apparent irrespective of the presence of SMuRFs. The majority of specific T‐cell subpopulations associated with CAD were within the differentiated cell compartment, apart from the naïve T‐cell subsets that showed a reciprocal reduction. Expansion of differentiated T‐cell subpopulations occurs during inflammation, and maintenance of an inflammatory state over a prolonged period can lead to a stable increase in the numbers of differentiated T cells. For Treg cells, increased numbers are indicative of the natural feedback loop present in virtually all immune inflammatory states.^24^, ^26^ While Treg dysfunction may also contribute mechanistically to development of atherosclerosis, the increase in cellular markers of Treg activation and differentiation – regardless of functionality – may prove to be a helpful measure of the disease process.

Reviewing the cell subset results in more detail, five CD4 conventional T‐cell subsets were found to be significantly different in CAD^+^ patients. We identified a reduction in naïve CD4 Tconv cells and an increase in CD45RO^+^CCR7^+^ effector memory (TEM) CD4 Tconv cells in those with CAD. These results are consistent with previously reported changes in the proportions of naïve and memory CD4 T cells in CAD.^27^, ^28^ CAD^+^ patients had increased percentages of CD4 Tconv cells expressing CCR2 and CCR4, chemokine receptors that indicate T‐cell differentiation. The CCR2 receptor has been shown to be causally involved in the generation of atherosclerosis in mouse knockout models,^29^ and at the genetic level, single‐nucleotide polymorphisms in the reading frame of CCR2 have been associated with an increased risk of myocardial infarction.^30^, ^31^ CCR2 is highly expressed by monocytes and binds to CCL2 which is primarily secreted by monocytes, macrophages and dendritic cells, supporting a primary role for monocyte/macrophage activation in atherosclerosis. To date, CD4 T‐cell subpopulations expressing CCR2 have received relatively little attention in relation to CAD in humans, as it has generally not been included in the focused studies of CD4 T‐cell phenotype. The chemokine receptor CCR4 binds multiple CC chemokines associated with inflammation, including CCL2, CCL4, CCL5, CCL17 and CCL22. It has historically been thought to be involved in allergy and asthma biology.^32^ The literature has little to say about CCR4 and atherosclerosis clinically, with only one brief report that described a positive correlation between CCR4 expressing CD4 T cells and Gensini score in a population of patients undergoing invasive angiography.^33^ The KLRG1^+^CD27^−^ subset was also found to be increased in the CD4 T‐cell compartment of CAD^+^ patients. This subset can alternatively be identified by means of granzyme B and perforin co‐expression and/or loss of CD28. None of these markers were included in our panel. Published reports have indicated that CD28^null^CD4 T cells are associated with CAD, especially in the context of CMV infection.^34^, ^35^, ^36^ Future studies will be required to confirm whether KLRG1^+^CD27^−^ cell numbers show an association with CMV, independent of CMV status.

Within CD8 T cells, three subsets were found to be significantly different in CAD^+^ patients. Circulating naïve CD8 T cells were less frequent in the CAD^+^ patients, consistent with previously reported findings in humans.^37^, ^38^ The KLRG1^+^CD27^−^ subset was also found to be increased in the CD8 T‐cell compartment. This CD8 T‐cell subset resembles the CD4 KLRG1^+^CD27^−^ subset in that it comprises highly differentiated cells that also express perforin, granzyme B, CD57, no longer express CD28, and have strong cytotoxic activity. Expression of CD56, another correlate of cytotoxic activity in CD8 T cells, was also increased in the CD8 T‐cell CAD^+^ cohort. The increase in highly differentiated and cytotoxic cell subsets within the CD8 T‐cell compartment in patients with CAD is in keeping with other studies which have demonstrated associations between ‘senescent’ cytotoxic CD8 T cells and arterial stiffness,^39^ hypertension,^40^ and higher short‐term mortality following heart attack.^41^

While our CD4 Tconv and CD8 T‐cell results are interesting, agree with the existing literature, and make biological sense, none persisted as statistically significant after adjustment for age and sex. Further study into patients with CAD incorporating adjustment of results for age may help clarify which of these T‐cell results pertain to atherosclerosis biology and which relate to more general age‐related changes, as the control populations used in this type of research are often significantly younger than the diseased population.

In our study, only three non‐T‐cell subsets were found to be significantly different in CAD^+^ patients. Circulating pDCs were found to be lower in the CAD^+^ group which agrees with previous findings in the literature.^42^, ^43^ pDCs are believed to drive pro‐atherogenic T‐cell immunity *via* antigen presentation on MHCII,^44^ though in our study, the association did not survive adjustment for age and sex. We demonstrated higher levels of CXCR3^+^ B cells and Ki67^+^ B cells in patients with CAD, and both of these relationships persisted as significant after adjustment for age and sex. CXCR3 is a chemokine receptor which is has primarily been studied in atherosclerosis in the context of T cells and NK cells, with Th1 cells expressing high levels of CXCR3 seen in atheroma and CXCR3 knockout mice having reduced atherosclerotic burden and lower levels of infiltrating T cells.^45^ In the B‐cell context, studies have demonstrated higher levels of circulating CXCR3 expressing B cells in patients with inflammatory disorders, such as rheumatoid arthritis,^46^ giant cell arteritis and polymyalgia rheumatica.^47^ To our knowledge, no previous study has described an association between CXCR3^+^ B cells and CAD. The significance of proliferating Ki67^+^ B cells in atherosclerosis is also not well understood. B‐cell research in human atherosclerosis has demonstrated a protective role for B1 cells and disease association with increased B2‐cell‐derived plasmablasts, with the majority of their effects mediated through production of antibodies and cytokines.^48^ B cells have been shown to demonstrate persistence of Ki67 expression for several days following completion of mitosis, making it difficult to distinguish between bone marrow‐derived populations and B cells undergoing peripheral proliferation.^49^ More detailed study into the phenotypes of circulating B cells associated with CAD^+^ will be required to clarify the importance of these cell types in atherosclerosis biology.

Finally, seven Treg cell subsets were found to be significantly increased in CAD^+^ patients, though the subsets expressing CCR6 and CD39 became non‐significant after adjustment for age and sex. In recent years, T regulatory phenotyping has been increasingly shown to identify specific immune signatures and predict responses to treatment.^18^, ^50^ In the atherosclerosis sphere, animal models have underpinned discovery work that has demonstrated that Tregs are present in atherosclerotic lesions and that their absence is associated with disease progression *via* multiple mechanisms, including anti‐inflammatory cytokine secretion and suppression of pro‐atherogenic effector T cells.^51^, ^52^ Much current research focuses on strategies that might target Tregs therapeutically in the future.^53^

In our study, the chemokine receptors found to be significantly associated with CAD^+^ in Tregs were CCR2, CCR4 and CCR6. The biology of CCR2 and CCR4 are discussed in the CD4 Tconv section above. In the Treg context, CCR2, CCR4 and CCR6 have previously been shown to be up‐regulated in Tregs migrating to non‐lymphoid tissues or sites of inflammation,^54^ and this may be the primary basis of their increase in the atherosclerosis phenotype. To our knowledge, CCR2‐ and CCR6‐expressing Treg subsets have not been specifically studied in relation to atherosclerosis. One previous study on atherosclerosis in mice may relate to CCR4‐expressing Tregs; it demonstrated that dendritic cell‐derived CCL17 drove atherosclerosis by restricting T regulatory cell homeostasis.^55^

We also identified a higher proportion of CD‐39 expressing Treg cells in CAD^+^ subjects. CD39 is an immunosuppressive ATPase expressed by functionally active CD45RO^+^ Tregs^56^ that also express CCR4. CD39 is one of the few T‐cell surface molecules for which a significant proportion of the population carries a low‐expressor allele.^57^ However, the low‐expressor phenotype appears to have only a subtle effect on overall immune function. In the present study, ~21% of subjects manifested the low‐expressor phenotype (Figure 3) and there was no significant enrichment of low expressors in either CAD^−^ or CAD^+^ subjects.

The proportions of Treg cells in the CAD^+^ group were also higher in those expressing HLA‐DR, Ki67, or co‐expressing CD38 and CD45RO, all of which are associated with activation of Treg cells. CD38 is a multi‐functional extracellular enzyme with NADase and cyclase activities which regulates T‐cell functions by metabolising NAD and affecting NAD‐related enzymes such as SIRT1, which is also of interest in cancer biology^58^ and is a treatment target for multiple myeloma.^59^ To our knowledge, there have been no studies performed which examined CD38^+^ CD45RO^+^ Treg cells in the context of atherosclerosis. HLA‐DR expression in Tregs has been shown to identify a mature and functionally distinct cell population which is involved in early contact‐dependent immune suppression *in vitro*,^60^ and while HLA‐DR expression throughout atheroma and on T cells is well described,^61^ no studies have specifically studied HLA‐DR expressing Tregs in the context of atherogenesis. Lastly, the Ki67^+^ Treg subset was increased in those with CAD, another marker which has been associated with Treg activation,^62^ though again the subset has not been studied specifically in association with atherosclerosis development.

Additionally, it is worth specifically commenting on the monocyte results. Multiple previous studies have shown significant increases in various CD16‐expressing monocyte populations in human CAD,^11^, ^12^, ^14^, ^15^, ^16^ and we expected to see similar results in our study. However, the resolution of CD16 expression was suboptimal in our study, and our findings are thus unreliable. The proportion of CD16^+^ monocytes was highly variable in the under 55 cohort, and restriction to the more homogenous subjects aged 55 years and above did not reveal any CAD associations. Another consideration is that our CAD subgroup includes individuals with less advanced disease compared to those studied in most of the monocyte literature, where disease was often defined as severe, multi‐vessel CAD. The monocyte results in our study will require further assessment in future experiments.

With the expected heterogeneity among large patient cohorts, the challenge in predictive modelling is to identify effective signatures for selected subpopulations rather than the entire patient population, a critical component in precision medicine. Our sub‐cohort analysis of the classifiability of patients initially showed that models combining the CyTOF markers performed worse in individuals who were less than 55 years of age. We then identified a signature that was predictive of CAD status in patients who were aged 55 and over, a finding that was confirmed in a smaller validation cohort. These results demonstrate that we can identify differences in circulating immune cell populations in the peripheral blood of patients with CAD compared to those with non‐diseased coronary arteries on CTCA, providing proof of concept for the use of advanced immune phenotyping as a potential diagnostic tool in the future. Interestingly, the differences in circulating immune cell populations were also apparent in SMuRFless CAD patients. Our findings are consistent with previous studies demonstrating the key role of inflammation in the pathophysiology of atherosclerosis and suggest that inflammation may lead to atherosclerosis even in the absence of traditional risk factors.

This study had several strengths, namely, that it included a large number of human patients with CTCA‐quantified CAD and utilised an unbiased approach to determine which cell populations were most important with respect to the presence or absence of CAD. We were able to employ a second validation experiment to confirm the replicability of our findings, though one limitation of the study was the lower power in that validation cohort. However, we applied a robust modelling approach which demonstrated that the identified correlation between the immune signature and the presence of CAD persisted for both cohorts. These interesting results will be evaluated further, using additional data drawn from the BioHEART‐CT cohort in the future.

While this study aims to examine differences in immune subpopulations seen in association with CAD, future efforts will focus on the potential for immune signatures to be used for early identification of CAD risk. This is of particular interest in light of the shared signature in CAD^+^ patients with and without SMuRFs. Our application of expert manual gating as the primary data analysis tool will ensure ready translation into laboratory tests based on standard gating of conventional fluorescence flow cytometry data. Further study is also required to establish whether distinct CAD phenotypes (e.g. calcified *vs* non‐calcified or vulnerable plaque) are associated with different immune profiles. Given the key role of inflammation in active atherosclerotic disease activity and patient outcomes, translation of these findings to a clinically useful tool would address a key unmet need in early identification and preventative treatment of CAD.

In conclusion, mass cytometry has provided an unbiased assessment of a multitude of immune cell subsets to assess those that are changed in the presence of CAD. This hypothesis‐generating experimental study has confirmed previous results, including the association of CAD with lower numbers of naïve CD4 and CD8 T cells and increases in cytotoxic CD4 and CD8 T cells. Our multiparametric approach also revealed many novel findings, including higher proliferation of T regulatory cells and B cells, and higher numbers of differentiated conventional and regulatory CD4 T cells. Future work in this cohort will be focused on determining whether these changes in cell proportions will inform cardiovascular risk profiling, better enabling precision diagnosis of coronary atherosclerosis, and potentially pointing us in the direction of new targeted therapies.

## Methods

### Study design

This study utilised samples from the BioHEART‐CT biobank (Australia New Zealand Clinical Trials Registry: ANZTR12618001322224, Northern Sydney Local Health District Human Research Ethics Committee approval: HREC/17/HAWKE/343). The study protocol has been described in detail previously.^63^ In brief, patients referred for clinically indicated CTCA who were aged 18 or older and who could provide informed consent were eligible. Patients were excluded if they were unwilling or unable to participate in phone follow‐up.

Patient demographic and clinical information were collected at the time of recruitment *via* questionnaire. Clinical information included past medical history, medication history, smoking history and any family history of CAD.

A discovery cohort of 132 patients from the BioHEART‐CT study who had PBMCs in frozen storage with cell numbers of 1 million or greater were selected for study. Fifteen samples were excluded from the discovery analysis because of technical issues during the experiment, insufficient cell viability on thawing or the presence of abnormally expanded cell populations because of haematological conditions that substantially altered cell population percentages, resulting in inclusion of 117 patients. A further 58 patients were selected for a validation experiment using the same criteria (Supplementary figure 7).

### Imaging acquisition and analysis

Computed tomography coronary angiography were acquired on a 256‐slice scanner using standard clinical protocols, overseen and dual‐reported by accredited cardiologists and radiologists. Heart rate was optimised using oral metoprolol or ivabradine based on body weight, as clinically appropriate. Prospective studies were preferentially performed unless heart rate control was suboptimal, in which case retrospective acquisition was utilised. Vasodilation was achieved using sublingual nitroglycerine (600–800 μg) given immediately prior to intravenous contrast delivery. Radiation doses were minimised as per guidelines,^64^ and reconstructions were performed using vendor‐specific software. CTCAs were analysed using the validated 17‐segment the Gensini score to identify those with and without CAD.^65^

### Biological samples and analysis

Peripheral venous blood samples were collected following insertion of a peripheral venous cannula for the CTCA. Blood was transferred into sodium citrate pathology tubes and kept at room temperature until processed. PBMCs were isolated within 4 h of blood collection using a standard gradient‐separation Ficoll–hypaque preparation.^66^ In brief, blood from the lithium heparin tube was diluted 1:1 with Hanks' Balanced Salt Solution (HBSS), layered onto Ficoll‐Paque Plus and centrifuged at 22°C at 1460 *g* without brake for 20 min. The layer containing the buffy coat was then washed twice with HBSS and centrifuged as above. The purified PBMCs were then immediately frozen in bovine serum (heat inactivated, Gibco, Australia) containing 10% dimethyl sulphoxide and were stored in liquid nitrogen.

Mass cytometry time‐of‐flight (CyTOF) was performed on the patient PBMCs. Cryopreserved samples were thawed in batches of ~20 in a 37°C water bath and pre‐warmed RPMI 1640 supplemented with 10% FCS and DNase (1:10 000). A panel of 41 metal‐tagged monoclonal antibodies supplied by the Ramaciotti Facility for Human Systems Biology Reagent Bank was used for analysis (Supplementary table 1). Unlabelled antibodies were purchased by the Ramaciotti Facility in a carrier‐protein‐free format, conjugated with the indicated metal isotope using the MaxPAR antibody conjugation kit (Fluidigm, San Francisco, USA), pre‐titred and provided in per‐test volumes. For live‐dead cell distinction, PBMCs were stained with 1.25 μm cisplatin. Cells were then incubated for 30 min initially with anti‐CD45 antibodies conjugated to various metals to generate barcodes. A barcoded reference PBMC internal control^67^ was then added to every sample before staining with the 35 remaining antibodies targeting surface antigens, with CD1c detected with anti‐CD1c‐PE followed by a ^156^Gd‐metal‐tagged anti‐PE antibody. Cells were then fixed and permeabilised using a FoxP3 staining kit (eBioscience, San Diego, USA) and stained with five intracellular antibodies (Supplementary table 1). Cells were fixed in 4% paraformaldehyde containing DNA intercalator (0.125 μm iridium‐191/193; Fluidigm) at room temperature for 20 min, then refrigerated overnight. After multiple washes, cells were diluted in MilliQ water containing 1:10 diluted EQ beads (Fluidigm) and acquired at a rate of 200–400 cells/s using a CyTOF 2 Helios upgraded mass cytometer (Fluidigm). All Helios data were normalised using the processing function within the CyTOF acquisition software based on the concurrently run EQ four element beads. Data analysis was performed using FlowJo version 10.4 software (FlowJo, LLC, Ashland, OR, USA). Samples were pre‐gated to exclude beads, dead cells and doublets and differentially barcoded cells were exported for further analysis.

### Statistical analysis

#### Descriptive statistics

Categorical variables are presented as frequencies and percentages, normally distributed continuous variables as means with standard deviations and non‐normally distributed continuous variables as medians with interquartile ranges. Those with a positive Gensini score were considered to have CAD (CAD^+^), and those with a Gensini score of zero were classified as being free of CAD (CAD^−^).

#### Differential proportion analysis, visualisation and validation

Cell type proportions were defined as either a proportion of total live cells (%total) or as a proportion of the relevant cellular subpopulation (%subpopulation). Clustering in the heatmap was performed on the cell type %totals of all patients, between proportions and between patients, using hierarchical clustering with Euclidean distance and complete linkage implemented in the R function *hclust*.

Uniform Manifold Approximation and Projection (UMAP) was performed using the runUMAP function in R package ‘scater’.^68^ All markers were used for clustering cells, with the following settings applied; n_neighbours = 15 and n_components = 2.

Significant differences in cell type proportions between CAD^−^ and CAD^+^ patients were assessed *via* logistic regression models where CAD^−^/CAD^+^ was the dependent variable. For each of the cell types, a model was constructed using the cell type proportion as the only independent variable, as well as a model including the cell type proportion, age and gender as independent variables. Associations were quantified as odds ratios with 95% confidence intervals.

The fold change of the mean of each cell type proportion between CAD^−^ and CAD^+^ patients was calculated for the discovery and validation cohorts of patients separately. A test for association of fold change between the two sets of patients was then performed using Pearson's correlation coefficient, to determine concordance between the two independent datasets.

#### Sub‐cohort signature model, validation and visualisation

Sub‐cohort analysis was performed by modelling the classifiability (i.e. the individual‐level accuracy rate) of an individual under a cross‐validation setting.^69^ A repeated five‐fold cross‐validation with 20 repeats was applied to the CyTOF data for all patients in the discovery cohort using a least absolute shrinkage and selection operator (lasso) logistic regression model, with the top 18 features (cell type proportions) selected by AUC in each fold before modelling. The patient classifiability was then measured for each patient by taking the proportion of correct predictions across each of the repetitions. To determine the modifying variables associated with patient classifiability, we regressed the individual classifiability score against the corresponding clinical risk factors using linear regression. The top modifying risk factors – age and statin – were identified using the significance of a non‐zero coefficient in the regression. Visualisation of the association between the individual classifiability score and the modifying risk factors showed that age 55 years or older was an appropriate cut‐off value to determine the cohort with higher classifiability scores.

Subsequent modelling was performed, and a radial kernel support vector machine (SVM) was determined to be the best performing model to build a risk model on patients who were age 55 or older (Discovery Cohort *n* = 88, Validation Cohort *n* = 41). The features included were CyTOF cell type proportions that were significant using the logistic regression models. Evaluation of the SVM risk model was performed both in the discovery and validation cohorts. Within the discovery cohort, a repeated five‐fold cross‐validation with 20 repeats with the top 18 features (of a total of 82) selected by AUC in each fold before modelling was performed, with the receiver‐operator curve (ROC) and AUC averaged over each fold. The SVM model was then trained on the whole discovery cohort using the 18 significant CyTOF cell populations from the logistic regression models, and the ROC and AUC calculated on the validation cohort.

To visualise the decision boundary of the SVM model using a principal component (PC) graph, the values of the principal components for the validation cohort were derived using the PC loadings from the discovery cohort. The grid of the PC graph was then mapped to the original data dimensions using a neural network trained on the discovery cohort, using the first two principal components as input variables. The neural network output was then used as input in the CyTOF CAD signature SVM model to obtain a prediction and create the decision boundary plot.

All data were analysed in R version 4.1^70^ and visualised using *ggplot2*.^71^

## AUTHOR CONTRIBUTIONS

**Katharine A Kott:** Conceptualization; data curation; formal analysis; investigation; methodology; project administration; writing – original draft; writing – review and editing. **Adam S Chan:** Data curation; formal analysis; methodology; visualization; writing – review and editing. **Stephen T Vernon:** Conceptualization; investigation; methodology; project administration; writing – review and editing. **Thomas Hansen:** Conceptualization; methodology; project administration; writing – review and editing. **Taiyun Kim:** Data curation; methodology; visualization; writing – review and editing. **Macha de Dreu:** Data curation; investigation; methodology; project administration; writing – review and editing. **Bavani Gunasegaran:** Data curation; formal analysis; investigation; methodology; writing – review and editing. **Andrew J Murphy:** Conceptualization; validation; writing – review and editing. **Ellis Patrick:** Formal analysis; methodology; software; supervision; validation; visualization; writing – review and editing. **Peter J Psaltis:** Conceptualization; validation; writing – review and editing. **Stuart M Grieve:** Conceptualization; investigation; methodology; supervision; validation; writing – review and editing. **Jean Y Yang:** Methodology; resources; software; supervision; writing – review and editing. **Barbara Fazekas de St Groth:** Conceptualization; data curation; formal analysis; funding acquisition; investigation; methodology; resources; supervision; writing – review and editing. **Helen M McGuire:** Conceptualization; data curation; formal analysis; funding acquisition; investigation; methodology; project administration; resources; supervision; visualization; writing – review and editing. **Gemma A Figtree:** Conceptualization; funding acquisition; investigation; methodology; project administration; resources; supervision; validation; writing – review and editing.

## Disclosures

GAF reports personal consulting fees from CSL and grants from Abbott Diagnostic outside the submitted work. In addition, GAF has a patent ‘Biomarkers and Oxidative Stress’ awarded USA May 2017 (US9638699B2) issued to Northern Sydney Local Health District. The other authors have no disclosures.

## Conflict of interest

The authors declare no conflict of interest.

## Supporting information


Supporting Information

## Data Availability

The data that support this study are available from the corresponding author upon reasonable request.
