# Supplementary material for: Mass cytometry analysis reveals altered immune profiles in patients with coronary artery disease
Source: Clin Transl Immunology. 2023 Nov 2;12(11):e1462. doi: 10.1002/cti2.1462 (PMC10621005; doi:10.1002/cti2.1462)
Supplement: Supplementary file 1 — Supporting Information [file CTI2-12-e1462-s001.docx]

# Supplementary Materials

Supplementary table 1 ………………………………………… Pages 2 and 3

Supplementary table 2 ………………………………………… Page 4

Supplementary table 3 ………………………………………… Page 5

Supplementary figure 1 ….……………………………………. Pages 6 and 7

Supplementary figure 2 ………………………………………. Page 8

Supplementary figure 3 ………………………………………. Pages 9 and 10

Supplementary figure 4 ………………………………………. Pages 11 and 12

Supplementary figure 5 ………………………………………. Pages 13 and 14

Supplementary figure 6 ………………………………………. Page 15

Supplementary figure 7 ………………………………………. Page 16

**Supplementary table 1**. Monoclonal antibodies used in this study. All metal-tagged antibodies were conjugated at the Ramaciotti Facility for Human Systems Biology collaborative initiative, in August 2020 or earlier.

| **Metal Isotope**  **/Fluorochrome** | **Antigen** | **Clone** | **Manufacturer of Unlabeled Antibody** | **†Signal**  **Discrimination** |
| --- | --- | --- | --- | --- |
|  | Barcoding and fluorescent | | | |
| 104Pd, 106Pd or 108Pd | CD45 | H130 | BD/Biolegend | Excellent |
| PE | CD1c | L161 | Biolegend | Excellent |
|  | Remaining surface stain | | | |
| 89Y | CD8a | RPA-T8 | BD | Excellent |
| 113In | CD56 | NCAM16.2 | BD | Good |
| 115In | CD11c | Bu15 | Biolegend | Excellent |
| 141Pr | P2X7 | L4 | In-house | Suboptimal |
| 142Nd | CD19 | HIB19 | Biolegend | Excellent |
| 143Nd | CD45RA | HI100 | Biolegend | Excellent |
| 144Nd | CD86 | IT2.2 | Biolegend | Excellent |
| 145Nd | CD4 | RPA-T4 | Biolegend | Excellent |
| 146Nd | IgD | IA6-2 | BD | Excellent |
| 147Sm | CD20 | 2H7 | Biolegend | Excellent |
| 148Nd | CD16 | 3G8 | BD | Suboptimal on monocytes |
| 150Nd | KLRG1 | SA231A | Biolegend | Excellent |
| 151Eu | CD39 | A1 | Biolegend | Excellent |
| 152Sm | CD45RO | UCHL1 | Biolegend | Good |
| 153Eu | CD304 | 12C2 | Biolegend | Excellent |
| 154Gd | CD196 | 11A9 | BD | Suboptimal on T cells |
| 155Gd | CD27 | M-271 | BD | Excellent |
| 156Gd | PE | PE001 | Biolegend | Excellent |
| 158Gd | CD194 (CCR4) | L291H4 | Biolegend | Excellent |
| 159Tb | CD197 (CCR7) | 150503 | R&D Systems | Suboptimal |
| 160Gd | CD14 | M5E2 | BD | Excellent |
| 161Dy | CD141 | AD5 | Miltenyi Biotec | Excellent |
| 163Dy | CD183 (CXCR3) | REA232 | Miltenyi Biotec | Suboptimal |
| 164Er | CD253 (TRAIL) | RIK-2 | Biolegend | Not detected |
| 165Ho | CD61 (Integrin B3) | VI-PL2 | Biolegend | Excellent |
| 166Er | CD34 | 581 | BD | Excellent |
| 167Er | CD38 | HIT2 | Biolegend | Excellent |
| 169Tm | CD25 | M-A251 | Biolegend | Excellent |
| 170Er | CD3 | UCHT1 | Biolegend | Excellent |
| 172Yb | CD192 (CCR2) | K036C2 | Biolegend | Excellent |
| 173Yb | Integrin beta 7 | FIB504 | BD | Excellent |
| 174Yb | HLA-DR | L243 | Biolegend | Excellent |
| 175Lu | LOX1 | 331212 | Life Technologies | Excellent |
| 176Lu | CD127 | A019D5 | Biolegend | Excellent |
| 209Bi | CD11b | ICRF44 | Biolegend | Excellent |
|  | Intracellular stain | | | |
| 139La | eNOS | polyclonal | R&D Systems | Not detected |
| 149Sm | NOX5 | polyclonal | Abcam | Suboptimal |
| 162Er | FOXP3 | PCH101 | eBioscience | Suboptimal |
| 168Er | Ki67 | B56 | BD | Excellent |
| 171Yb | Arginase 1 | 14D2C4 | Biolegend | Suboptimal |
| 191/193Ir | DNA Intercalator | - | Fluidigm | Excellent |

† Signal discrimination is classified according to the degree to which the reagent allowed for a clear, binary distinction. Excellent: bimodal expression pattern with clear distinction between the two peaks; Good: bimodal expression pattern with distinction between the two peaks requiring expert estimation of gate placement; Suboptimal: expression pattern lacking clear bimodality, with estimation of gate placement based on expression above the level in known negative subpopulations; Not detected: reagent positive for specific cell types such as *in vitro* activated cells and tumours, but no expression detected in PBMCs in this study.

| **Demographics, Risk Factors & Medications** | **Discovery**  **n = 117** | **Validation**  **n = 58** | **p** |
| --- | --- | --- | --- |
| Sex, female – n (%) | 57 (49%) | 28 (48%) | 0.956 |
| Age, years – mean (SD) | 61 (12) | 61 (12) | 0.702 |
| BMI, kg/m^2^ – mean (SD) | 27.1 (4.8) | 27.4 (5.7) | 0.766 |
| Hypertension – n (%) | 50 (43%) | 27 (47%) | 0.632 |
| Diabetes – n (%) | 8 (7%) | 6 (10%) | 0.421 |
| Hyperlipidaemia – n (%) | 62 (53%) | 37 (64%) | 0.175 |
| Current smoking – n (%) | 13 (11%) | 2 (3%) | 0.088 |
| Significant Smoking History (> 10 pack years) – n (%) | 23 (20%) | 12 (21%) | 0.872 |
| Patients with no major risk factors (SMuRFless) – n (%) | 23 (20%) | 6 (10%) | 0.119 |
| Significant Family History of Ischaemic Heart Disease – n (%) | 33 (28%) | 11 (19%) | 0.185 |
| Antiplatelet – n (%) | 23 (20%) | 13 (22%) | 0.671 |
| Statin – n (%) | 38 (33%) | 26 (45%) | 0.110 |
| Beta-blocker – n (%) | 19 (16%) | 11 (19%) | 0.652 |
| ACE-inhibitor / Angiotensin Receptor Blocker – n (%) | 41 (35%) | 24 (41%) | 0.414 |
| CAD^+^ - n (%) | 79 (68%) | 28 (48%) | 0.014 |
| Gensini score for CAD^+^ – median (IQR) | 7.5 (13.5) | 13.3 (30) | 0.296 |

**Supplementary table 2**: Validation cohort characteristics; coronary artery disease, CAD; standard deviation, SD; body mass index, BMI, standard modifiable risk factors, SMuRFs, angiotensin converting enzyme, ACE; interquartile range, IQR. *P*-values calculated using the Wilcoxon rank sum test for continuous variables; Fisher’s exact test for categorical variables with all expected cell counts < 5; Pearson’s Chi-squared test for categorical variables with any expected cell count ≥ 5.

**Supplementary table 3**: Characteristics of the easy and hard to classify sub-cohorts within the discovery cohort; coronary artery disease, CAD; standard deviation, SD; interquartile range, IQR. *P*-values calculated using Wilcoxon rank sum test for continuous variables; Fisher’s exact test for categorical variables with all expected cell counts < 5; Pearson’s Chi-squared test for categorical variables with any expected cell count > = 5.

| **Demographics, Risk Factors & Medications** | **Hard**  **(Age < 55)**  **n = 33** | **Easy**  **(Age** $\boldsymbol{\geq}$ **55)**  **n = 84** | **p** |
| --- | --- | --- | --- |
| Sex, female – n (%) | 12 (36%) | 45 (54%) | 0.094 |
| Age, years – mean (SD) | 45 (7) | 67 (7) | <0.001 |
| BMI, kg/m^2^ – mean (SD) | 27.3 (4.5) | 27.0 (5.0) | 0.834 |
| Hypertension – n (%) | 12 (36%) | 38 (45%) | 0.383 |
| Diabetes – n (%) | 5 (15%) | 3 (4%) | 0.026 |
| Hyperlipidaemia – n (%) | 15 (46%) | 47 (56%) | 0.306 |
| Current smoking – n (%) | 5 (15%) | 8 (10%) | 0.383 |
| Significant Smoking History (> 10 pack years) – n (%) | 6 (18%) | 17 (20%) | 0.801 |
| Patients with no major risk factors (SMuRFless) – n (%) | 11 (33%) | 12 (14%) | 0.020 |
| Significant Family History of Ischaemic Heart Disease – n (%) | 12 (36%) | 21 (25%) | 0.219 |
| Antiplatelet – n (%) | 4 (12%) | 19 (23%) | 0.199 |
| Statin – n (%) | 6 (18%) | 32 (38%) | 0.038 |
| Beta-blocker – n (%) | 1 (3%) | 18 (21%) | 0.015 |
| ACE-inhibitor / Angiotensin Receptor Blocker – n (%) | 10 (30%) | 31 (37%) | 0.501 |
| CAD+ - n (%) | 15 (46%) | 64 (76%) | 0.001 |
| Gensini score – median (IQR) | 0 (7) | 3.5 (12) | <0.001 |


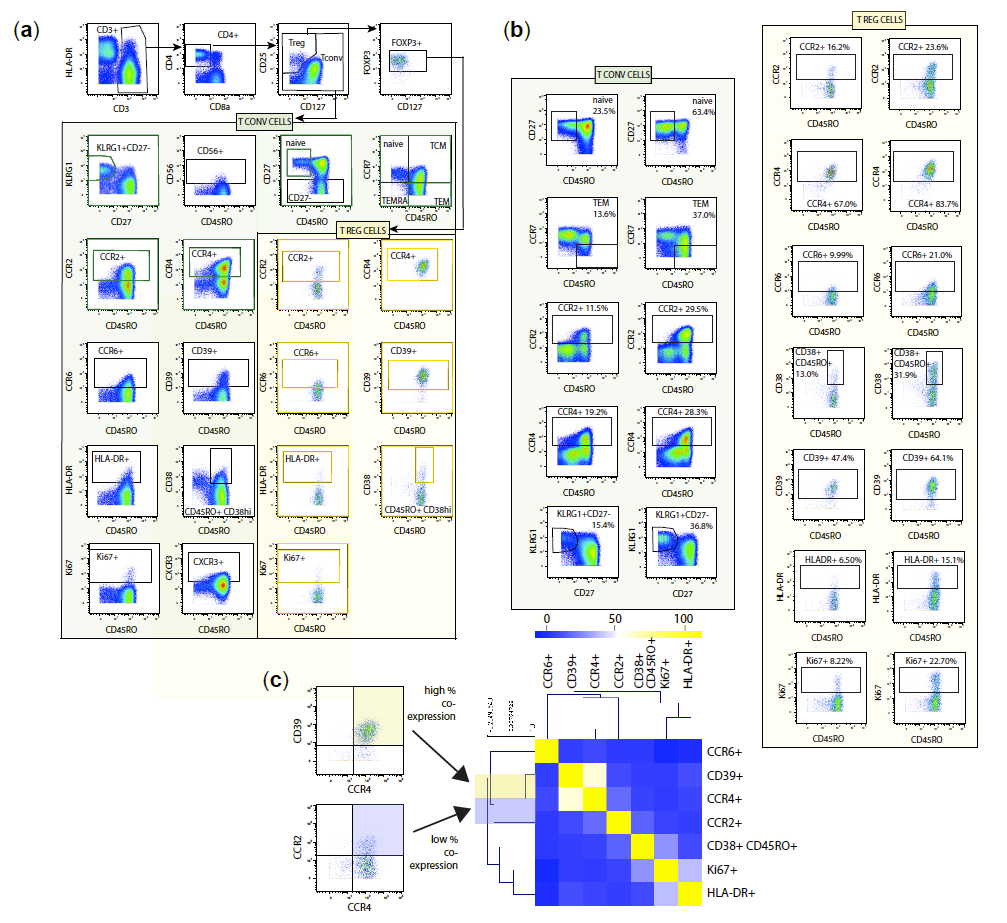


**Supplementary figure 1**: (**a**) Gating tree for CD4 T cells showing the subdivision between CD127^lo^CD25^+^ Tregs and the remaining Tconv. Populations with significantly different proportions (between CAD^-^ and CAD^+^) within Tconv or Tregs are highlighted with green and yellow outlines, respectively. Note the suboptimal signals for CCR6 and CXCR3, as documented in Supplementary table 1. (**b**) Examples of the significant populations in individual subjects with relatively low (left hand column) or high (right hand column) proportions, demonstrating clear, bimodal distributions of marker expression, with the exception of CCR6^+^ Tregs. (**c**) Examples of marker expression on Tregs as highly correlated (top) or not (bottom), with correction matrix across all markers assessed showing two distinct modules of relation: (i) CD39 and CCR4 and (ii) Ki67^+^ HLA-DR^+^.


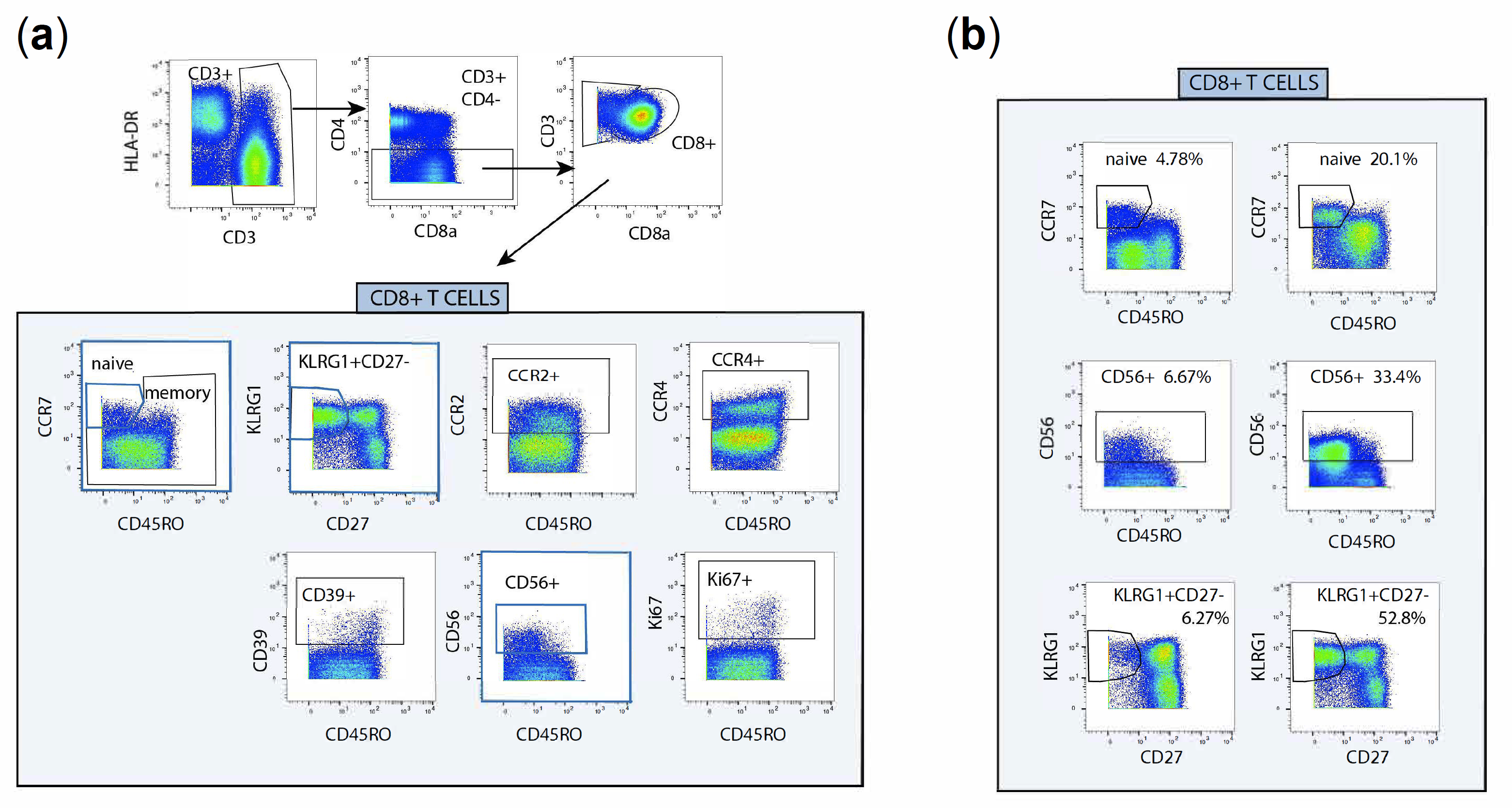


**Supplementary figure 2**: (**a**) Gating tree for CD8 T cells. Populations with significantly different proportions (between CAD^-^ and CAD^+^) within CD8 T cells are highlighted in blue. (**b**) Examples of the significant populations in individual subjects with relatively low (left hand column) or high (right hand column) proportions, demonstrating clear, bimodal distributions of marker expression.


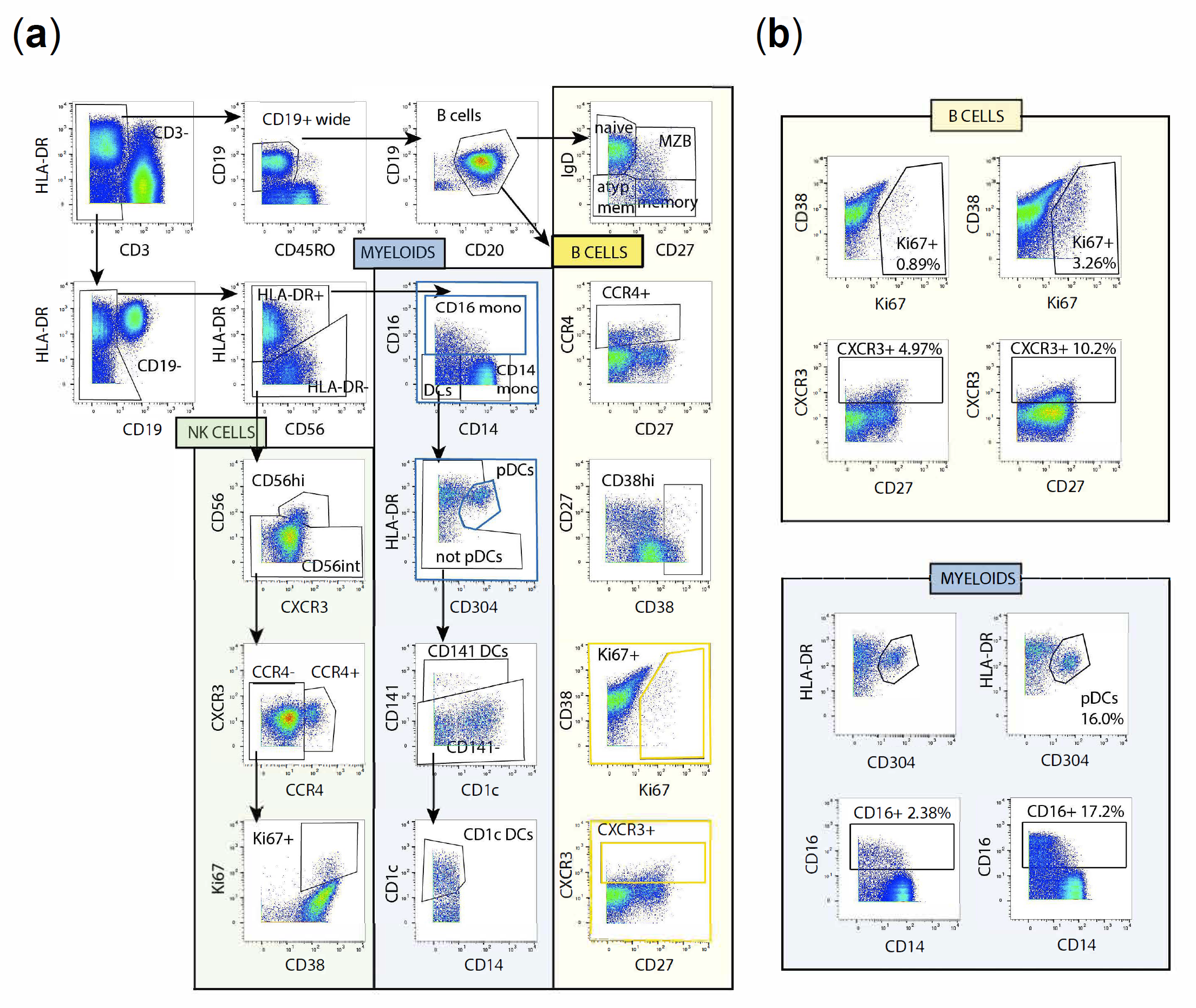


**Supplementary figure 3**: (**a**) Gating tree for non-T cells. Populations with significantly different proportions within B cells and myeloid cells are highlighted in yellow and blue, respectively. No NK cell populations were significantly different between CAD^-^ and CAD^+^ subjects. Spill over of CD38 into the Ki67 channel was compensated for by the polygonal gate applied to NK and B cells, both of which express uniformly high levels of CD38. (**b**) Examples of the significant B cell and myeloid populations in individual subjects with relatively low (left hand column) or high (right hand column) proportions. Although the CXCR3 signal was suboptimal, the difference between the gated percentages within B cells in CAD^-^ versus CAD^+^ subjects was statistically significant. CD16^+^ monocyte numbers were highly variable, with no statistically significant differences between CAD^-^ and CAD^+^ subjects (*P* = 0.236) in subjects 55 years and above.

**
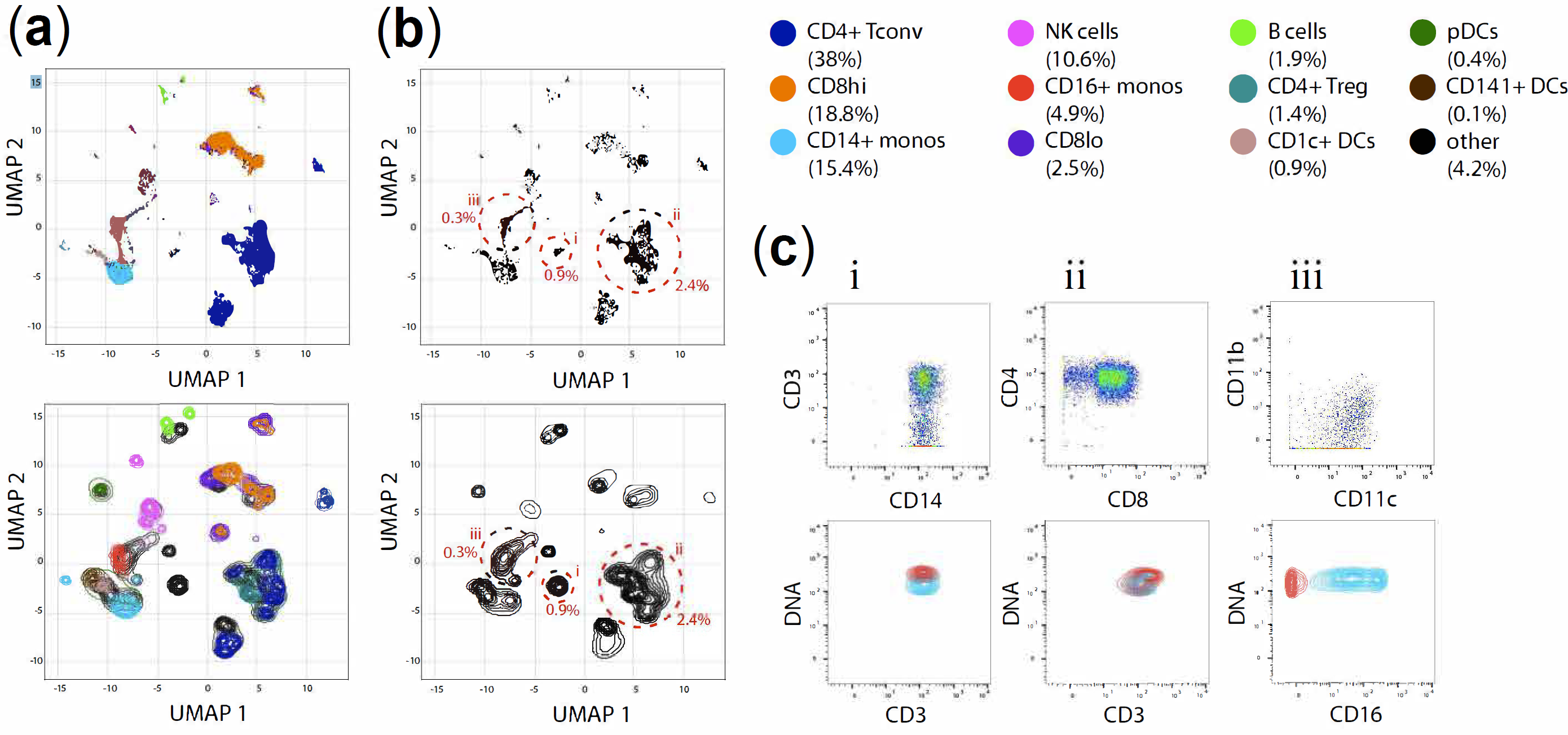
**

**Supplementary figure 4: UMAP visualisations demonstrating high degree of correlation with traditional gating strategies.** (**a**) UMAP visualisation showing overlayed dot-plots (top row) and 5% contour line demarcation (bottom row), showing the 11 major immune populations in colour as gated across Supplementary figures (accounting for 95.8% of cells), with residual non-gated cells in black (“other”, 4.2%). (**b**) UMAP visualisation of the 4.2% “other” cells, with the 3 largest clusters labelled i-iii) gated for further analysis. (**c**) (i–iii) dot plots (upper panel) and 5% contour plots of DNA intercalator, a surrogate for cell size (lower panel) of the three gated populations in **b**. Cluster (i) ~70% of this cluster expresses both CD3 and CD14, indicating doublets between T cells and monocytes. Overlay contours of cluster (i) in red vs CD4 T cells in blue indicate that cluster (i) is composed entirely of doublets. Cluster (ii) is mainly composed of doublets. Cluster (iii) is likely to be a myeloid population expressing CD11c but low for CD11b and CD16, likely due to a staining problem. Overlay contours of cluster (iii) in red vs CD16 monocytes in blue indicate that they are of similar size, consistent with a myeloid origin.

**
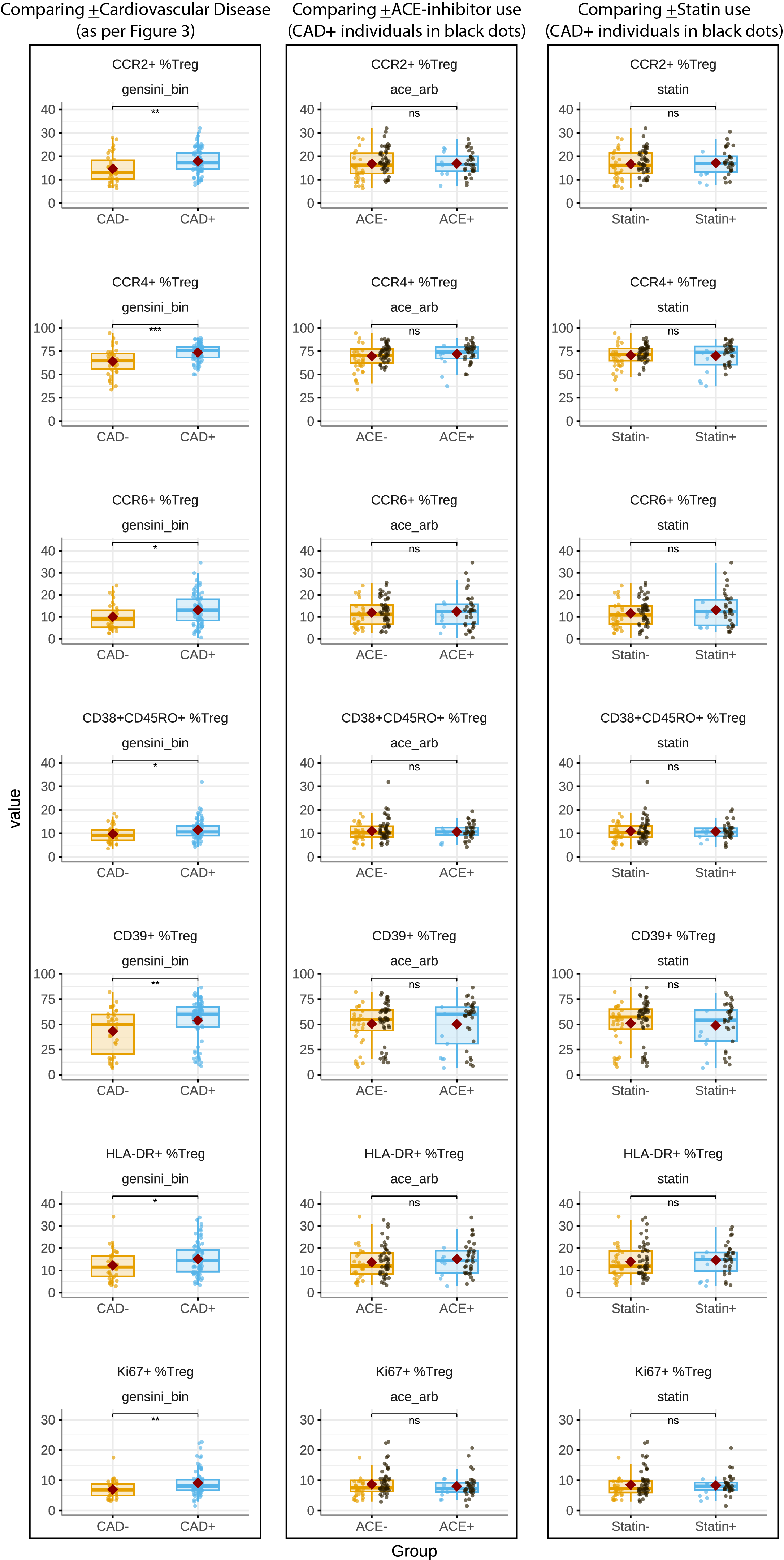

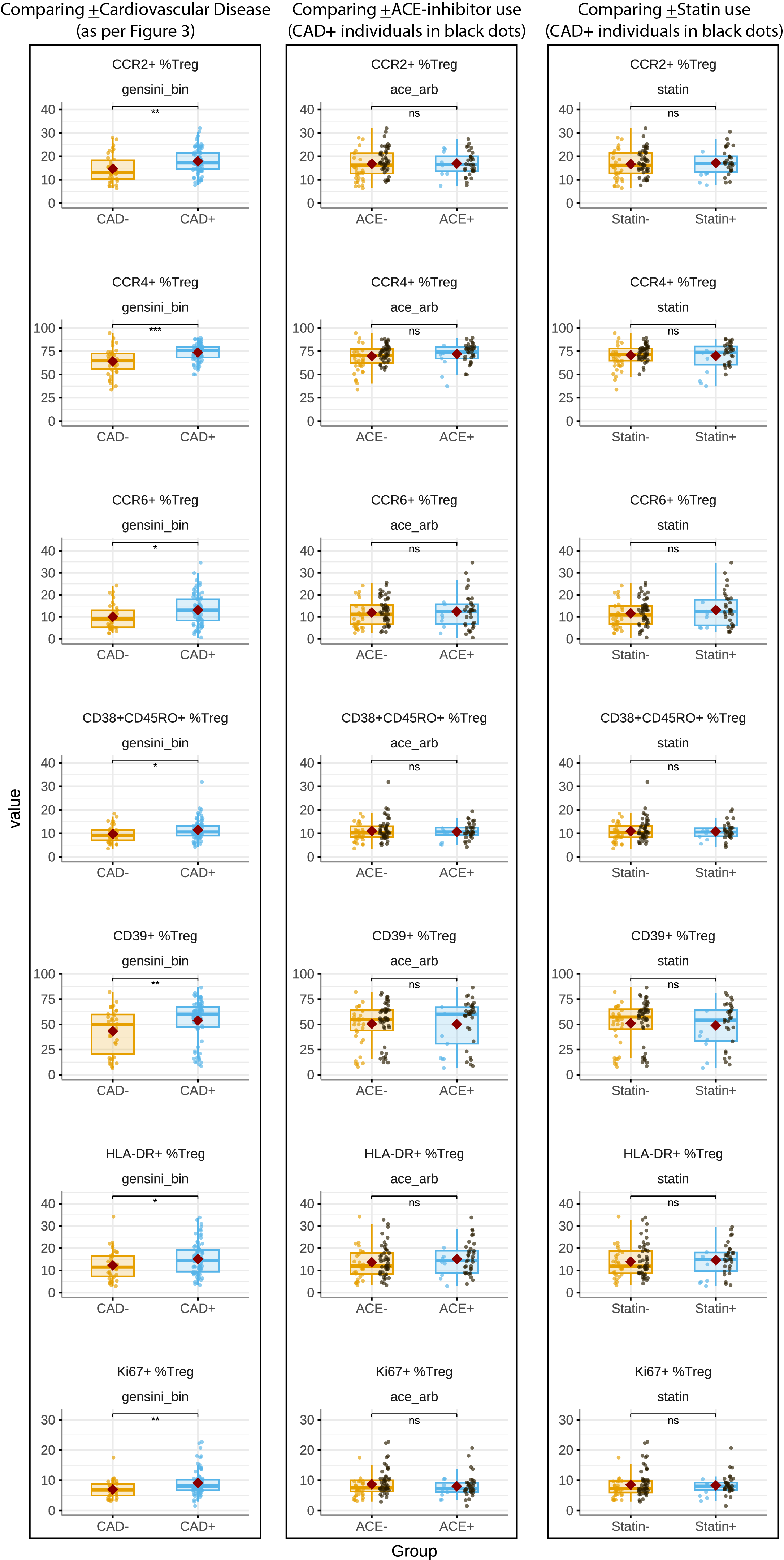
**

**Supplementary figure 5:** Boxplots showing the proportions of selected cellular subtypes of T regulatory cells (Tregs) comparing: **left column)** non-diseased (CAD^-^) and diseased (CAD^+^) individuals; **middle column)** ACE-inhibitor usage; and **right column)** statin medication usage, with CAD^+^ individuals for middle and right columns shown in black. *P-*values were calculated using the Wilcoxon test; n = 177. * *P* < 0.05, ** *P* < 0.01, *** *P* < 0.001.


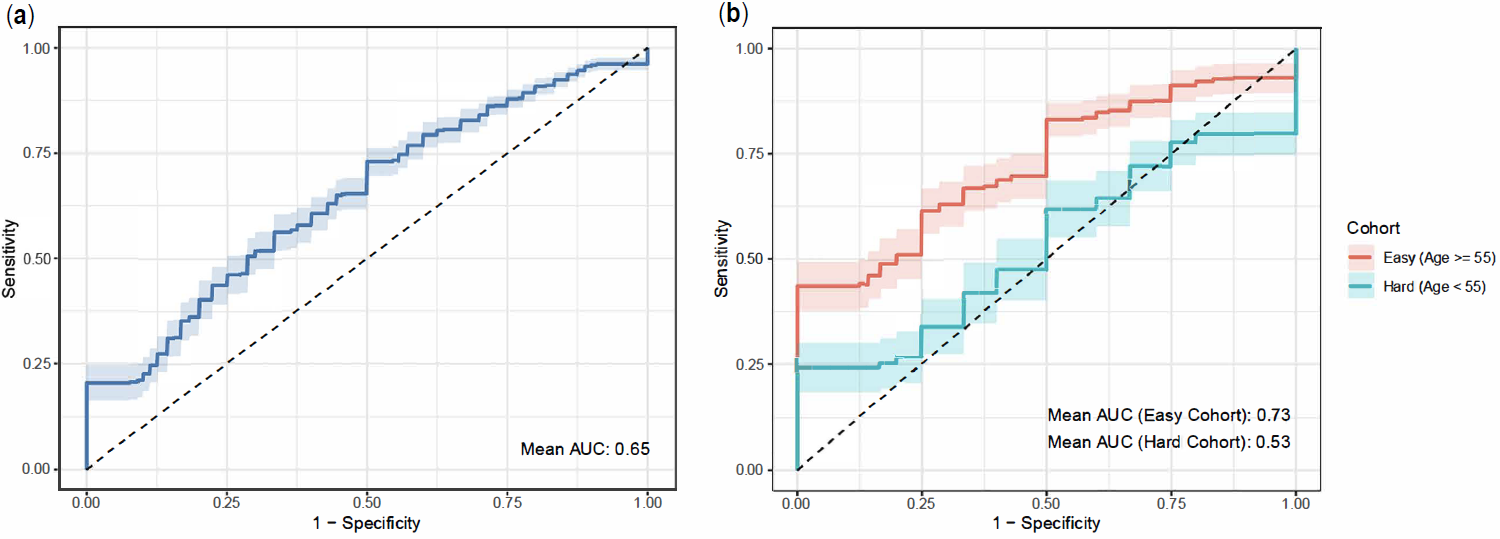


**Supplementary figure 6**: Receiver operating characteristic curves, averaged over a 5-fold cross validation with 20 repeats within: (**a**) the whole discovery cohort (n = 117), and (**b**) the discovery cohort separated into sub-cohorts split by age (“Easy” n = 33, “Hard” n = 84).


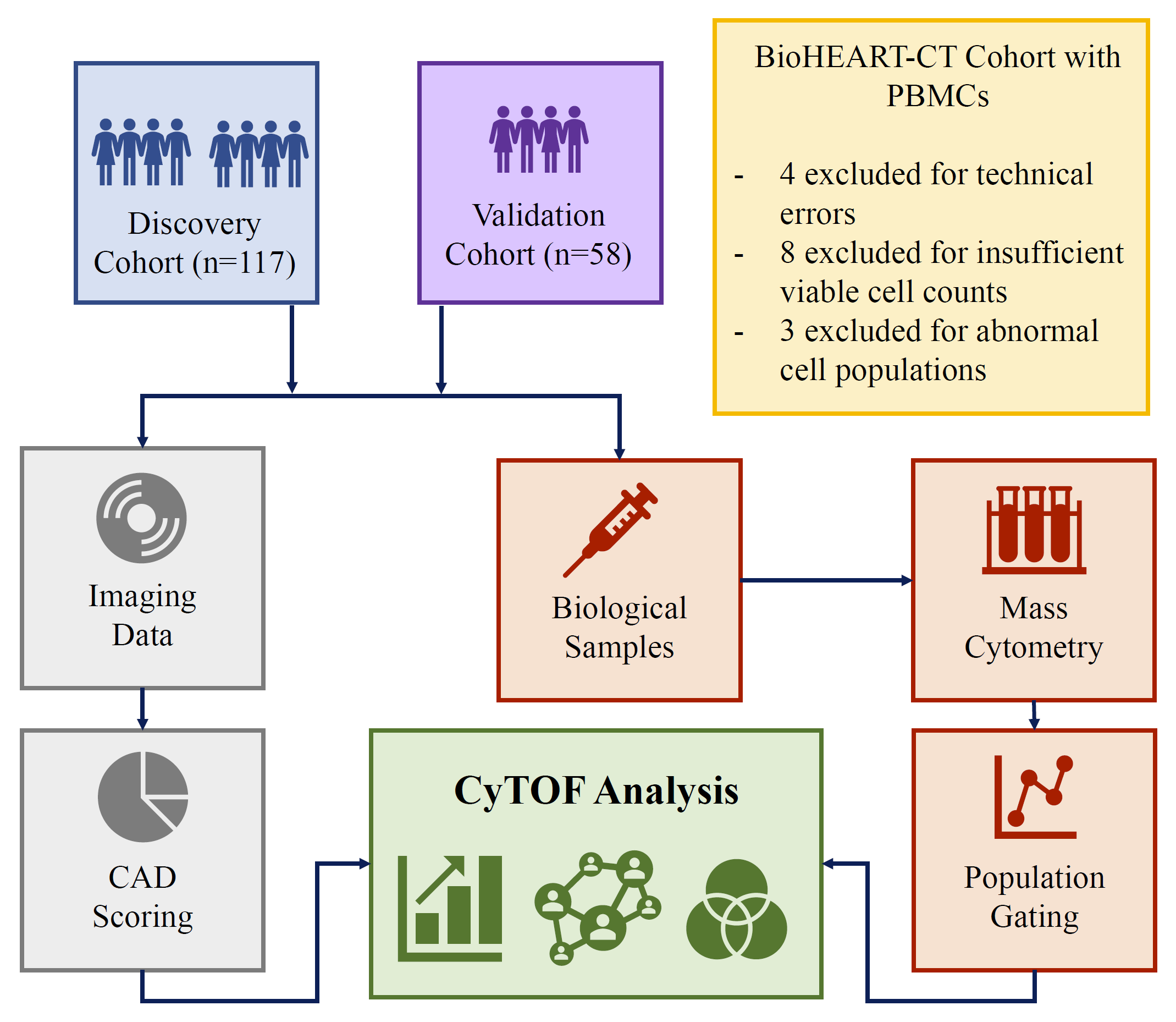


**Supplementary figure 7**: Diagram of Study Design; coronary artery disease, CAD; mass cytometry time of flight, CyTOF; peripheral mononuclear blood cells, PBMCs.
